# Ceftazidime-avibactam alone or in combination with Aztreonam versus Polymyxins in the management of carbapenem-Resistant *Klebsiella pneumoniae* nosocomial Infections (CAPRI study): a retrospective cohort study from South India

**DOI:** 10.1007/s15010-023-02094-9

**Published:** 2023-09-11

**Authors:** Racha Amarthya Sree, Anand Gupta, Nitin Gupta, Sadhana Veturi, L. Siva Kumar Reddy, Masrath Begum, Etrouth Shravani, HariPriya Reddy Challa, Satti Santhosh Reddy, Adarsh Singamsetty, Murthy Arumilli, P. Naveen Reddy, Praveen Kumar Tirlangi

**Affiliations:** 1 G. Pulla Reddy College of Pharmacy, Hyderabad, Telangana India; 2 Department of Critical Care Medicine, AIG Hospitals, Hyderabad, Telangana India; 3 Department of Infectious Diseases, Kasturba Medical College Manipal, Manipal Academy of Higher Education, Manipal, Karnataka 576104 India; 4 Department of Microbiology, AIG Hospitals, Hyderabad, Telangana India; 5 RBVRR Women’s College of Pharmacy, Hyderabad, Telangana India; 6 Department of Clinical Pharmacy, AIG Hospitals, Hyderabad, Telangana India; 7 Department of Infectious Diseases, AIG Hospitals, Hyderabad, Telangana India; 8 Department of Clinical Pharmacy, AIG Hospitals, Hyderabad, India; 9 Critical Care Medicine, AIG Hospitals, Hyderabad, Telangana India; 10 Department of Medicine, AIG Hospitals, Hyderabad, Telangana India

**Keywords:** Carbapenem-resistant *Klebsiella pneumoniae*, Ceftazidime–avibactam, Polymyxin, Colistin, NDM-New Delhi Metallo-beta-lactamase

## Abstract

**Introduction:**

Carbapenem-resistant *Klebsiella pneumoniae* (CRKP) infections commonly cause hospital-acquired infections. The study aimed to compare the outcomes of CRKP infections between patients receiving ceftazidime avibactam +/− aztreonam and polymyxins in a hospital setting with a high prevalence of New Delhi Metallo Beta Lactamase production.

**Methods:**

We conducted a retrospective cohort study from January 2020 to September 2022 in critically ill adult patients admitted to a non-COVID-19 medical intensive care unit with CRKP infection**.** The patients were followed up for a total of 30 days or death, whichever was later.

**Results:**

Of a total of 106 patients included in the study, 65 patients received polymyxins and 41 patients received ceftazidime–avibactam +/− aztreonam. Higher 30-day mortality was noted in the polymyxin group (56.9% vs. 29.2%, *P* = 0.005). The mean time to event (mortality) in ceftazidime–avibactam +/− aztreonam was 23.9 + 1.5 days which was significantly higher compared to polymyxins (17.9 + 1.2 days, *p* = 0.006). On Cox regression analysis, after adjusting for the covariates, the hazard ratio for time to event with the use of polymyxin was 2.02 (95% CI: 1.03–3.9).

**Conclusion:**

Ceftazidime–avibactam + aztreonam is possibly associated with better clinical outcomes in patients infected with CRKP.

## Background

Carbapenem-resistant *Klebsiella pneumoniae* (CRKP) infections are a common cause of hospital-acquired infections in endemic regions. Traditionally, polymyxins with or without other antibiotics are used for the treatment of CRKP infections, but their use is associated with poor outcomes [1, 2]. Besides, polymyxin use is also associated with significant toxicity [3]. Ceftazidime–avibactam is a newer beta-lactam–beta-lactamase inhibitor (BL–BLI) used for the treatment of Gram-negative infections [4]. Its efficacy has been shown to be comparable to carbapenem in patients with infections susceptible to both drugs [5–7]. It is also effective against CRKP as it is active against the two main carbapenemases, OXA-48 and KPC [4]. In India, carbapenem resistance in CRKP is primarily mediated by OXA-48 and the New Delhi Metallobetalactamases (NDM) [8]. Ceftazidime–avibactam is not active against NDM CRKP. Aztreonam is a monobactam antibiotic that is active against NDM, but it can be hydrolyzed by other bacterial enzymes which are often co-existent with NDM in CRKP [9]. Therefore, a novel strategy of a synergistic combination of ceftazidime–avibactam and aztreonam has been suggested to treat NDM CRKP infections [10]. To the best of our knowledge, there is very limited literature on the real-world use of this synergistic combination. The study aimed to compare the outcomes of ceftazidime–avibactam + aztreonam with polymyxins.

## Methodology

### Study design and recruitment

This was a retrospective cohort study (January 2020–September 2022) conducted in a tertiary-care hospital in South India after Institute Ethics Committee approval (AIG/IEC-Post BH&R 36/11.2022–01). All consecutive critically ill adult patients diagnosed with a nosocomial infection caused by CRKP infection were treated with either ceftazidime–avibactam with or without aztreonam or polymyxins (polymyxin B or colistin). Critically ill were defined as those admitted to the medical intensive care units (exclusively for non-COVID-19 patients). Nosocomial infection was defined as an infection acquired after 48 h of admission and was classified as blood-stream infections (BSI), hospital-acquired pneumonia (HAP), urinary tract infection (UTI), and skin–soft-tissue infection (SSTI) based on the involvement of the respective organ systems. HAP was defined as new onset dyspnoea or tachypnoea with chest infiltrates and respiratory specimens growing CRKP. Primary BSI was defined as the isolation of CRKP from the blood with compatible symptoms of bacteremia, such as fever, rigours, or hypotension. When BSI was associated with the simultaneous presence of CRKP infection at other sites (respiratory tract, urinary tract, and skin/soft tissue), they were deemed as secondary BSI. UTI was defined as the presence of urinary symptoms such as dysuria and increased frequency or fever with isolation of CRKP from urine samples. SSTI was defined as inflammation of the skin or soft tissue with isolation of CRKP from pus, exudates, or skin biopsy specimens.

CRKP infection was defined as the isolation of *Klebsiella pneumoniae* from blood, urine, respiratory specimens, or tissue, and proven resistance to meropenem or imipenem on susceptibility testing. In this study, only those patients with clinical symptomatology suggestive of infection of a particular site with positive culture were included. We did not include patients with poly-bacterial infections or those where colonization was suspected. Those patients who received antibiotics for less than 48 h or who died within 48 h of receiving antibiotics were excluded. Since 48 h is not enough for the antibiotics to act and, therefore, it can be assumed that their impact on mortality will be minimal. Those patients who received both the study antibiotics were also excluded.

## Diagnosis and treatment

Blood, respiratory specimens (Bronchoalveolar lavage, Endotracheal aspirate), and urine and tissue specimens were collected for BSI, HAP, UTI, and SSTI, respectively. These samples were sent for culture and susceptibility testing. Identification of *Klebsiella pneumoniae* and susceptibility testing was done by the Vitek 2 Compact System (Biomerieux, France). The isolates were labelled as resistant as per the clinical breakpoints set by the Clinical and Laboratory Standards Institute. Synergy testing was done in those isolates that were resistant to ceftazidime–avibactam. Synergy testing was done using a double-disk diffusion test between ceftazidime–avibactam and aztreonam using previously described methods [11].

The routine practice was to start the empiric therapy within 1 h of clinical diagnosis in those patients where a nosocomial infection was suspected. Empiric therapy for nosocomial infections was usually combination therapy with polymyxin [polymyxin B (12,500 units/kg every twelve hours) or colistin (4.5 million international units every 12 h)]. The empiric therapy was either continued, or they were changed to ceftazidime–avibactam (2.5 g eighth hourly) with/without aztreonam (2 g eighth hourly). Adjunctive antibiotics, such as tigecycline, fosfomycin, carbapenems, and minocycline, were added to the study antibiotics in some patients based on the physician’s choice. Adjunctive antibiotics were not exclusively guided by the susceptibility pattern. Both polymyxins and ceftazidime–avibactam were available in the hospital pharmacy during the study period. The choice of definitive therapy was based on the physician’s discretion. Patients with phenotypic susceptibility to ceftazidime–avibactam were treated with ceftazidime–avibactam alone. Patients with phenotypic resistance to ceftazidime–avibactam but a positive synergy with aztreonam were treated with a combination of ceftazidime–avibactam and aztreonam. Whenever used together, ceftazidime–avibactam and aztreonam were infused concurrently. The antibiotics (ceftazidime–avibactam, colistin, aztreonam, carbapenems, and fosfomycin) were dose-modified according to the renal function based on standard dosing nomograms (UpToDate, Waltham, MA). New onset liver or renal dysfunction, thrombocytopenia, and dialysis requirement after initiation of trial drugs were noted. Liver dysfunction was defined as transaminases more than three times the upper limit of normal (40 IU/L) if the baseline was normal or more than three times the baseline if it was abnormal. Renal dysfunction was defined as serum creatinine more than 1.5 times the upper limit of normal (1.2 mg/dl) if the baseline was normal or more than 1.5 times the baseline if it was abnormal. Thrombocytopenia was defined as a platelet count less than the lower limit of normal (150,000/microlitre).

### Follow-up and outcomes

The patients were followed up from receiving the first dose of the antibiotic in question for 30 days or mortality, whichever was later. The primary outcome was the all-cause 30-day mortality and the secondary outcome was the clinical cure. Clinical cure was defined as the resolution of fever with significant clinical improvement (decrease in sequential organ function assessment score by 2 points) in the condition of the patient on day 14. The records were assessed by infectious disease physicians to assess clinical cure.

### Data analysis

**Fig. 1:**
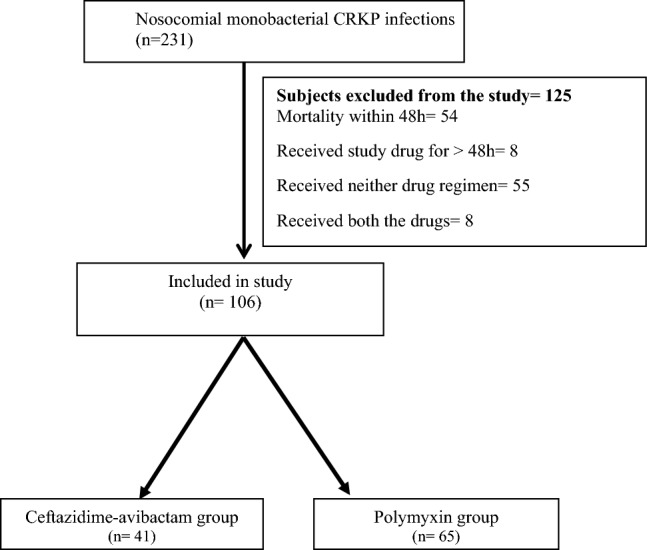
Recruitment of the patients and the study plan

The data were retrieved from electronic records by a team of clinical pharmacists, microbiologists, and infectious disease physicians. Using an alpha error of 0.05, a power of 80%, and the expected proportion of 30-day mortality of 44% and 20% in the polymyxin and ceftazidime–avibactam arm, a total sample size of 92 was calculated [12]. The patients were divided into two arms: ceftazidime–avibactam (with or without aztreonam) and polymyxin arm (polymyxin or colistin) (Fig. 1). The baseline characteristics of the two groups were compared. For categorical variables, a Chi-square test was used. For continuous variables, based on the normality of distribution, either an independent test was used, or a Mann–Whitney U test was used. The time to event (mortality) was compared between the two groups using the log-rank test. A Cox regression analysis was done using other covariates that could predict mortality to calculate the adjusted hazard ratio for the use of polymyxin. Those covariates were selected that have a higher risk of mortality based on the available literature. These were age, severe disease at presentation (SOFA score, vasopressor requirement, mechanical ventilation, PaO2/FiO2 ratio), HAP, and PBSI. A *p* value of < 0.05 was taken as significant.

## Results

A total of 106 patients were included in the study. All the 65 patients in the polymyxin arm were susceptible to polymyxin. The 41 patients in the ceftazidime–avibactam arm included 19 patients who were susceptible to ceftazidime–avibactam and, therefore, received ceftazidime–avibactam alone. The 22 patients who were resistant to ceftazidime–avibactam but susceptible to synergy testing received a combination of ceftazidime–avibactam with aztreonam. None of the tested isolates were resistant to synergy testing. Genotyping (Xpert Carba-R, Cepheid, Sunnyvale, CA) could be done in only 23 patients with the following results: NDM + OXA-48 (*n* = 15), OXA-48 alone (*n* = 6), NDM alone (*n* = 1), and no genes detected (*n* = 1). Thirty patients required renal dose modification, 20 of which were for the antibiotics used in the polymyxin arm.

The baseline characteristics, syndromic diagnosis, and clinical severity were comparable between the two groups (Table 1). Systemic corticosteroids in the recent past were not given prior to the development of infection in any of the patients. Only two patients had malignancy, both were in the polymyxin arm. There were no transplant recipients or patients with human immunodeficiency virus infection in the cohort. Adjunctive antibiotics were more commonly given along with polymyxins based on susceptibility pattern and physician’s choice (Table 2). There was no difference between the adverse drug reactions reported in both groups (Table 2). There was no difference in terms of clinical success between the two groups (Table 2). The 30-day all-cause mortality was noted to be significantly higher in the polymyxin group (Table 2).

**Table 1:** Comparison of baseline characteristics between ceftazidime–avibactam and polymyxin arms

Parameters		Ceftazidime–avibactam arm (*n* = 41)	Polymyxin arm (*n* = 65)	*p* value
Gender	Male	29 (70.7%)	51(78.4%)	0.368
	Female	12 (29.3%)	14(21.6%)	
Age (in years)		55.9 + 16.3	58.6 + 12.3	0.333
Comorbidities	Diabetes mellitus	22 (56.3%)	38(58.4%)	0.627
	Hypertension	24(58.5%)	37(56.9%)	0.87
	Coronary artery disease	9 (21.9%)	14((21.5%)	0.96
	Chronic liver disease	7 (17.0%)	12(18.4%)	0.856
	Chronic kidney disease	7(17.0%)	12(18.4%)	0.856
	Chronic lung disease	4 (9.7%)	5(7.6%)	0.710
Clinical severity*	Sequential Organ Function Assessment Score	5 (2–7.5)	6 (4–9)	0.056
	P/F ratio	249.5 + 132.3	221.9 + 99.4	0.254
	Mechanical ventilation	28 (68.2%)	51(78.4%)	0.242
	Shock leading to vasopressor requirement	17(41.4%)	32(49.2%)	0.435
	Dialysis requirement	7 (17%)	15 (23%)	0.458

^*^At the time of recruitment (initiation of the first dose of antibiotic in question)

**Table 2:** Comparison of clinical diagnosis, treatment details and outcomes between ceftazidime–avibactam and polymyxin arms

Parameters		Ceftazidime–avibactam arm (*n* = 41)	Polymyxin arm (*n* = 65)	*p* value
Clinical diagnosis	HAP	24(58.5%)	48(73.8%)	0.1
	UTI	4(9.7%)	6(9.2%)	0.928
	SSTI	2(4.8%)	2(3%)	0.636
	BSI	18(43.9%)	20(30.7%)	0.17
	Primary BSI	11 (26.8%)	12 (18.4%)	0.309
	Secondary BSI	7^#^ (17%)	8^$^ (12.3%)	0.493
Duration of hospitalization*		14 (8–18.5)	11 (7–17.5)	0.183
Percentage susceptibility to adjunctive antibiotics	Tigecycline	21(51.2%)	34 (55.7%)	
	Minocycline	2 (4.9%)	6 (9.8%)	
	Fosfomycin	10 (24.4%)	25 (41%)	
Adjunctive antibiotics	Any adjunctive antibiotic	19(46.3%)	52(80%)	< 0.001
	Any susceptible adjunctive antibiotics (excluding aztreonam)	11 (26.8%)^&^	33 (50.7%)^	0.014
	Tigecycline	9 (21.9%)	25 (38.4%)	0.076
	Carbapenem	5 (12.1%)	22 (33.8%)	0.013
	Minocycline	4 (9.7%)	9 (13.8%)	0.532
	Fosfomycin	5 (12.1%)	12 (18.4%)	0.392
Adverse drug reaction	Renal dysfunction	10 (24.4%)	20 (30.7%)	0.48
	Dialysis requirement	6 (14.6%)	9 (13.8%)	0.91
	Liver dysfunction	2 (4.9%)	3 (4.6%)	0.95
	Thrombocytopenia	8 (19.5%)	10 (15.4%)	0.58
Outcome	Clinical success	20 (48.7%)	20 (30.7%)	0.062
	Mortality	12 (29.2%)	37(56.9%)	0.005

Abbreviation- Hospital-acquired pneumonia (*HAP*), Urinary tract infection (*UTI*), Skin–soft-tissue infection (*SSTI*), Bloodstream infection (*BSI*)

^#^(Secondary to HAP-5, SSTI-2), ^$^(Secondary to HAP-7, UTI-1) ^&^(Tigecycline-6, Minocycline-2, Fosfomycin-3), ^(Tigecycline-19, Minocycline-3, Fosfomycin-11)

Of the 52 patients who received polymyxin combination therapy, there were 29 (56%) deaths. Of the 13 patients who received monotherapy with polymyxins, there were eight (61%) deaths. This difference was not significant (*p* = 0.7). In the patients enrolled in the polymyxin arm, there were 48 patients who received polymyxin B and 17 patients who received colistin. The mortality in patients who received colistin (8/17, 47%) was non-significantly lower than those who received polymyxin B (29/48, 60%), (*p* = 0.34)**.**

When the analysis was restricted to polymyxins vs. ceftazidime–avibactam and aztreonam, the mortality in the ceftazidime–avibactam and aztreonam combination arm [36% (8/22)] was lower than the polymyxin arm (57%, 37/65). This difference was, however, non-significant (*p* = 0.09). The mortality difference between ceftazidime–avibactam alone and ceftazidime–avibactam with aztreonam was not significant (*p* = 0.28).

The two most common syndromes in this study were HAP and BSI. The mortality in patients with HAP in the ceftazidime–avibactam group (10/24, 42%) was lower than in the polymyxin group (30/48, 62%) but the difference was statistically non-significant (*p* = 0.09). The mortality in patients with BSI in the ceftazidime–avibactam group (6/18, 33%) was lower than in the polymyxin group (13/20, 65%) but the difference was statistically non-significant (*p* = 0.051).

**Fig. 2:**
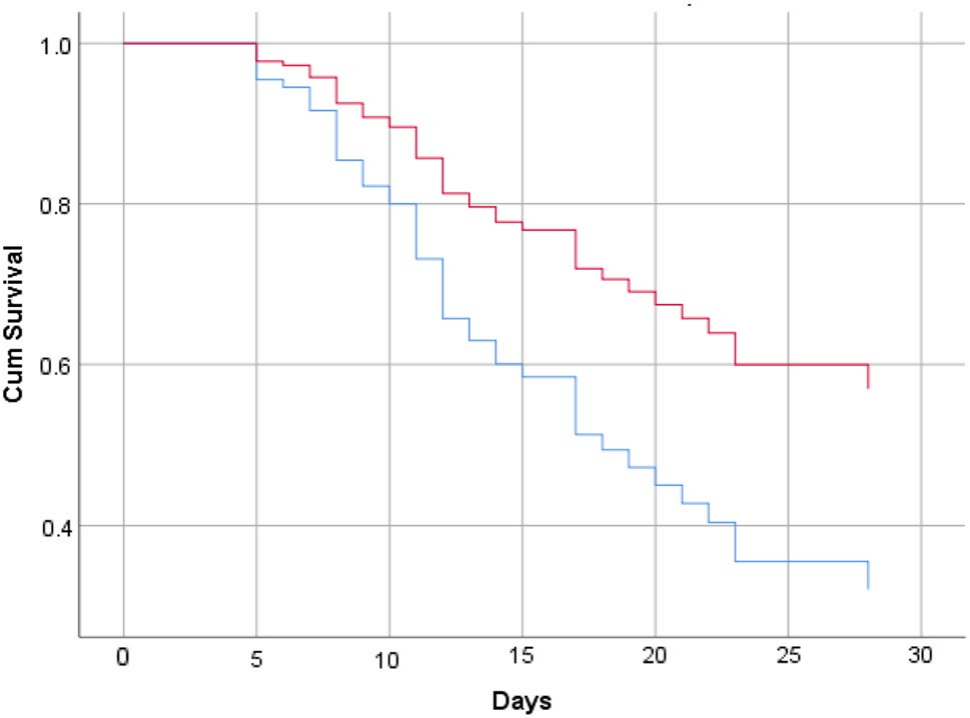
Survival curve showing time to mortality in patients treated with ceftazidime–avibactam + aztreonam (red line) vs polymyxin (blue line) adjusted for covariates

The mean time to event in ceftazidime–avibactam + aztreonam was 23.9 + 1.5 days which was significantly higher compared to polymyxins (17.9 + 1.2 days, *p* = 0.006, log-rank test) (Fig. 2). On Cox regression analysis, after adjusting for the covariates, the hazard ratio for time to event with the use of polymyxin was 1.97 (95% CI: 1–3.9) (Table 3).

**Table 3:** Cox regression analysis to determine the association between potential predictors and time to death

Predictors	Hazard ratio	95.0% CI		*p* value
		Lower	Upper	
Use of polymyxin	1.971	1.005	3.867	0.048
Age	1.029	1.003	1.055	0.028
PaO2/FiO2 ratio	.998	.995	1.001	0.244
Mechanical ventilation	1.647	.661	4.100	0.284
Vasopressor requirement	.848	.448	1.604	0.611
Sequential Organ Function Assessment score	1.160	1.068	1.261	< 0.001
Hospital-acquired pneumonia*	.793	.168	3.730	0.769
Primary blood stream infection	.972	.185	5.109	0.973

^*^Includes blood-stream infection secondary to hospital-acquired pneumonia

## Discussion

In patients admitted to a medical intensive care unit (for non-COVID-19 patients) with CRKP infections, all-cause 30-day mortality was significantly lower in the ceftazidime–avibactam arm when compared to polymyxins. The mortality difference between the two arms, when the analysis was restricted to patients with HAP or BSIs, was not significant. In the ceftazidime–avibactam arm, when patients who received both ceftazidime–avibactam and aztreonam were only considered, the mortality rate was still lower than polymyxins, but the difference was not statistically non-significant. The mortality difference between polymyxin B vs. colistin or polymyxin monotherapy vs. combination groups was not significant either.

CRKP infections are associated with significant morbidity and mortality. Polymyxins are the last resort of the antibiotics used in the management of these infections. The safety and efficacy of polymyxin drugs are compromised due to their poor pharmacokinetic properties and toxicity [3]. Ceftazidime–avibactam is a newer beta-lactam–beta-lactamase inhibitor that has efficacy against OXA-48 and KPC carbapenemases. Multiple studies in the past have proven the superior safety and efficacy of ceftazidime–avibactam compared to polymyxins in CRKP infections mediated by KPC and OXA-48 [12–19] (Table 4). We reviewed seven observational studies on CRKP infections (KPC or OXA-48) treated by ceftazidime–avibactam and polymyxins. The mortality with ceftazidime–avibactam ranged from 8 to 37%, whereas it was 30–70% for patients treated with polymyxin. All studies showed better mortality outcomes with ceftazidime–avibactam.

**Table 4:** Mortality associated with ceftazidime–avibactam and polymyxins in the previous studies on CRKP infections

S. no	Author	Study design	Sample size	Predominant carbapenemases	Outcome	Ceftazidime–avibactam	Polymyxins
1	Van Duin et al. [11]	Prospective observational study	137	KPC	30-Day all-cause mortality	8%	33%
2	Almangour et al. [12]	Retrospective observational cohort study	230	OXA-48	In-hospital mortality	35%	44%
3	Hakeam et al. [13]	Retrospective cohort study	61	OXA-48	30-Day mortality	37.5%	41.4%
4	Fang et al. [14]	Retrospective observational study	115	-	28-Day mortality	8.1%	29.5%
5	Castón et al. [15]	Retrospective observational study	339	OXA-48 and KPC		21.9%	46.9%
7	Satlin et al. [16]	Retrospective observational study	137	KPC	30-Day mortality	10%	31%
8	Falcone et al. [17]	Prospective observational study	102 patients	Metallo beta lactamases	30-Day mortality	19.2%	44%
9	Current study	Retrospective observational study	106	OXA-48 and NDM	30-Day mortality	29%	57%

It has been suggested in the recent past that ceftazidime–avibactam when combined with aztreonam can be used in patients with NDM infections [17]. This is particularly important for countries like India, where NDM carbapenemase production is the major mechanism of carbapenem resistance. Although we could not do genotyping in most of our patients, the presence of resistance to carbapenems and ceftazidime–avibactam in the 22 patients in one arm was used as a surrogate for the presence of NDM. These patients were then treated with ceftazidime–avibactam and aztreonam based on susceptibility observed on double-disk synergy testing. It must be noted here that synergy testing is a surrogate for the actual susceptibility testing of aztreonam–avibactam antibiotic.

As shown in Table 4, the study by Falcone et al. was the only major study that focused on NDM and compared the efficacy of ceftazidime–avibactam with aztreonam combination against polymyxins [12]. In our study, there was significantly lower overall 30-day mortality and time to event in the ceftazidime–avibactam arm when compared to polymyxins, and the results remained significant after adjusting for covariates. We chose 30-day all-cause mortality as our primary outcome. This was in line with the recommendations given by the consensus guidelines on VAP [20]. This is primarily because this is the most patient-relevant outcome. Since critically ill patients have a high risk of acquiring additional nosocomial infections, their 30-day mortality can still be higher even after recovering from a CRKP infection. In this regard, we wanted to see which drug is associated with a higher time to event. We did not choose attributable mortality as attributing mortality to a particular infection in a critically ill adult is very difficult and subjective, especially in a retrospective study. The findings in our study are similar to the experience obtained with studies on KPC and OXA (Table 3). The overall mortality was, however, very high in our cohort compared to the studies listed in Table 3. This was mostly because we included critically ill patients with high baseline severity. The disease severity in the ceftazidime–avibactam was lesser, but the difference was not significant. Nevertheless, the time to event between the two arms was adjusted for severity on Cox regression analysis. The comorbidities in the two arms were balanced. Charlson Comorbidity Index could not be calculated as most patient charts did not have definitive data on dementia and peptic ulcer disease. In our study, there was a difference in clinical success between the two arms. It was tending towards statistical significance. Since the definition of clinical success was more subjective than mortality, we kept clinical success as the secondary outcome.

Our results are different from a recently published study from South India that has shown no difference in mortality after introducing ceftazidime–avibactam ± aztreonam for the treatment of CRKP infections. However, it must be noted here that the study was conducted in two different timelines where improvement in supportive care could influence the results despite the adjustments [21].

There was no difference between the incidence of the new requirement of dialysis, new onset thrombocytopenia, renal dysfunction, or liver dysfunction between the ceftazidime–avibactam and the polymyxin arms in this study. We were not able to assess the neurological side effects in our study due to its retrospective design. Also, the high usage of adjunctive antibiotics in both groups makes it difficult to attribute a side effect to one particular antibiotic.

It is important to note here that the recommended method for polymyxin susceptibility testing is broth microdilution. The susceptibility testing for polymyxin in this study was done by the VITEK2 method which is not reliable [22, 23]. It is possible that CRKP isolates in the polymyxin arm were resistant in the control arm and this would explain the poor outcome. In an ideal scenario, the susceptibility of polymyxins should be available to guide treatment decisions. In resource-limited settings, polymyxin susceptibility by broth microdilution is rarely available, even in the best of centres. Similarly, genotyping for the mechanism of carbapenem resistance is not available in many centres.

Since both drug strategies have a poor evidence base, it is common practice to combine the antibiotics in question with adjunctive antibiotics. Combination therapy with adjunctive antibiotics was used in 71/106 (66.9%) patients in our study cohort. The most used antibiotic in combination with polymyxin and ceftazidime–avibactam was tigecycline. Since this was an observational study and the treatment decisions were not taken by the study investigators, 20% of patients received polymyxin monotherapy. The use of combination therapy, theoretically, can prevent the development of resistance as well [24]. In a recent meta-analysis based mostly on studies with a high risk of bias, polymyxin combination therapy was shown to fare better than monotherapy [25]. The use of monotherapy in our study, however, did not show statistically significant poorer outcomes when compared to polymyxin combination therapy. This was possibly because our study was underpowered to detect that difference.

In the polymyxin arm, 26% (*n* = 17) of the patients received colistin. Except for UTIs, polymyxin B is preferred over colistin because of its favourable nephrotoxicity profile and better pharmacokinetics/pharmacodynamics [26]. This is based on expert consensus recommendations only. It must be noted here that, in our study, there was no significant difference between the mortality outcomes of colistin and polymyxin B. This is in congruence with a recent meta-analysis that did not show any difference in outcomes between polymyxin B and colistin [27].

The most common syndrome included in this cohort was HAP. Since polymyxins have poorer penetration in the lungs, it can be argued that this might have contributed to worse outcomes in the polymyxin arm [28]. It must, however, be noted that, although, in patients with HAPs, there were fewer deaths in the ceftazidime–avibactam arm, the difference was statistically non-significant. Similarly, in patients with BSI, the difference in mortality between the two arms was not significant. These results were possibly because the study was underpowered to detect differences in these subgroups.

Our study has a few limitations. Being a retrospective study, the unknown confounding factors or confounding by indication could have influenced our study results. However, we also believe that there is such a paucity of Indian data on this subject, that retrospective studies such as this will serve as the stepping stone for future prospective clinical trials. At baseline, more patients in the polymyxin arm compared to ceftazidime–avibactam + aztreonam were on mechanical ventilation and required vasopressors; however, the difference was not statistically significant. Our results did not change after adjusting for these covariates. Genotyping could not be done in most patients and, therefore, the mechanism of carbapenem resistance could not be ascertained in all patients. Typing of strains could not be performed for the same reasons. Polymyxin susceptibility was not ascertained using the recommended broth microdilution method.

Despite these limitations, our study results suggest better efficacy of ceftazidime–avibactam in combination with aztreonam compared to polymyxins in the treatment of CRKP isolates in settings with a high prevalence of NDM carbapenemases. Further randomized-controlled studies are required to confirm our results.

## Data Availability

Data “available on request”.

## Abbreviations

CRKP: Carbapenem-Resistant Klebsiella Pneumoniae
NDM: New Delhi Metallo-beta-lactamase

## References

[1] Paul M, Daikos GL, Durante-Mangoni E, Yahav D, Carmeli Y, Benattar YD (2018). Colistin alone versus colistin plus meropenem for treatment of severe infections caused by carbapenem-resistant Gram-negative bacteria: an open-label, randomised controlled trial. Lancet Infect Dis.

[2] Kaye KS, Marchaim D, Thamlikitkul V, Carmeli Y, Chiu CH, Daikos G (2022). Colistin monotherapy versus combination therapy for carbapenem-resistant organisms. NEJM Evid..

[3] Wagenlehner F, Lucenteforte E, Pea F, Soriano A, Tavoschi L, Steele VR (2021). Systematic review on estimated rates of nephrotoxicity and neurotoxicity in patients treated with polymyxins. Clin Microbiol Infect.

[4] van Duin D, Bonomo RA (2016). Ceftazidime/Avibactam and Ceftolozane/Tazobactam: second-generation β-Lactam/β-lactamase inhibitor combinations. Clin Infect Dis.

[5] Wagenlehner FM, Sobel JD, Newell P, Armstrong J, Huang X, Stone GG (2016). Ceftazidime-avibactam Versus Doripenem for the treatment of complicated urinary tract infections, including acute pyelonephritis: RECAPTURE, a phase 3 randomized trial program. Clin Infect Dis Off Publ Infect Dis Soc Am.

[6] Mazuski JE, Gasink LB, Armstrong J, Broadhurst H, Stone GG, Rank D (2016). Efficacy and safety of Ceftazidime-Avibactam plus metronidazole versus meropenem in the treatment of complicated intra-abdominal infection: results from a randomized, controlled, double-blind, phase 3 program. Clin Infect Dis Off Publ Infect Dis Soc Am.

[7] Torres A, Zhong N, Pachl J, Timsit JF, Kollef M, Chen Z (2018). Ceftazidime-avibactam versus meropenem in nosocomial pneumonia, including ventilator-associated pneumonia (REPROVE): a randomised, double-blind, phase 3 non-inferiority trial. Lancet Infect Dis.

[8] Garg A, Garg J, Kumar S, Bhattacharya A, Agarwal S, Upadhyay GC (2019). Molecular epidemiology & therapeutic options of carbapenem-resistant Gram-negative bacteria. Indian J Med Res.

[9] Shields RK, Doi Y (2020). Aztreonam combination therapy: an answer to metallo-β-lactamase–producing gram-negative bacteria?. Clin Infect Dis Off Publ Infect Dis Soc Am.

[10] Marshall S, Hujer AM, Rojas LJ, Papp-Wallace KM, Humphries RM, Spellberg B (2017). Can ceftazidime-avibactam and aztreonam overcome β-lactam resistance conferred by metallo-β-lactamases in enterobacteriaceae?. Antimicrob Agents Chemother.

[11] European Committee on Antimicrobial Susceptibility Testing Antimicrobial Susceptibility Testing EUCAST Disk Diffusion Method Version 10.0. [(accessed on 28 August 2023)]. Available online: https://www.eucast.org/ast_of_bacteria/disk_diffusion_methodology

[12] Falcone M, Daikos GL, Tiseo G, Bassoulis D, Giordano C, Galfo V (2021). Efficacy of ceftazidime-avibactam Plus Aztreonam in patients with bloodstream infections caused by metallo-β-lactamase-producing *Enterobacterales*. Clin Infect Dis Off Publ Infect Dis Soc Am.

[13] van Duin D, Lok JJ, Earley M, Cober E, Richter SS, Perez F (2018). Colistin versus ceftazidime-avibactam in the treatment of infections due to carbapenem-resistant *Enterobacteriaceae*. Clin Infect Dis Off Publ Infect Dis Soc Am.

[14] Almangour TA, Ghonem L, Aljabri A, Alruwaili A, Al Musawa M, Damfu N (2022). Ceftazidime-avibactam versus colistin for the treatment of infections due to carbapenem-resistant *Enterobacterales*: A Multicenter Cohort Study. Infect Drug Resist.

[15] Hakeam HA, Alsahli H, Albabtain L, Alassaf S, Al Duhailib Z, Althawadi S (2021). Effectiveness of ceftazidime–avibactam versus colistin in treating carbapenem-resistant Enterobacteriaceae bacteremia. Int J Infect Dis IJID Off Publ Int Soc Infect Dis.

[16] Fang J, Li H, Zhang M, Shi G, Liu M, Wang Y (2021). Efficacy of ceftazidime-avibactam versus polymyxin b and risk factors affecting clinical outcomes in patients with carbapenem-resistant *Klebsiella* pneumoniae Infections a Retrospective Study. Front Pharmacol.

[17] Castón JJ, Cano A, Pérez-Camacho I, Aguado JM, Carratalá J, Ramasco F (2022). Impact of ceftazidime/avibactam versus best available therapy on mortality from infections caused by carbapenemase-producing Enterobacterales (CAVICOR study). J Antimicrob Chemother.

[18] Satlin MJ, Chen L, Gomez-Simmonds A, Marino J, Weston G, Bhowmick T (2022). Impact of a rapid molecular test for *Klebsiella* pneumoniae carbapenemase and ceftazidime-avibactam use on outcomes after bacteremia caused by carbapenem-resistant enterobacterales. Clin Infect Dis Off Publ Infect Dis Soc Am.

[19] Zheng G, Zhang J, Wang B, Cai J, Wang L, Hou K (2021). Ceftazidime-Avibactam in combination with in vitro non-susceptible antimicrobials versus ceftazidime-avibactam in monotherapy in critically ill patients with carbapenem-resistant *Klebsiella* pneumoniae infection: A Retrospective Cohort Study. Infect Dis Ther.

[20] Spellberg B, Talbot G (2010). Recommended design features of future clinical trials of antibacterial agents for hospital-acquired bacterial pneumonia and ventilator-associated bacterial pneumonia. Clin Infect Dis Off Publ Infect Dis Soc Am.

[21] Manesh A, Shankar C, George MM, Jasrotia DS, Lal B, George B (2023). Clinical and genomic evolution of carbapenem-resistant *Klebsiella* pneumoniae bloodstream infections over two time periods at a tertiary care hospital in south india: A Prospective Cohort Study. Infect Dis Ther.

[22] Khurana S, Malhotra R, Mathur P (2020). Evaluation of Vitek®2 performance for colistin susceptibility testing for Gram-negative isolates. JAC-Antimicrob Resist.

[23] Lo-Ten-Foe JR, de Smet AMGA, Diederen BMW, Kluytmans JAJW, van Keulen PHJ (2007). Comparative evaluation of the VITEK 2, disk diffusion, etest, broth microdilution, and agar dilution susceptibility testing methods for colistin in clinical isolates, including heteroresistant Enterobacter cloacae and Acinetobacter baumannii strains. Antimicrob Agents Chemother.

[24] Soman R, Veeraraghavan B, Hegde A, Jiandani P, Mehta Y, Nagavekar V (2019). Indian consensus on the management of CRE infection in critically ill patients (ICONIC) - India. Expert Rev Anti Infect Ther.

[25] Hou SY, Wu D, Feng XH (2020). Polymyxin monotherapy versus polymyxin-based combination therapy against carbapenem-resistant Klebsiella pneumoniae: a systematic review and meta-analysis. J Glob Antimicrob Resist.

[26] Tsuji BT, Pogue JM, Zavascki AP, Paul M, Daikos GL, Forrest A (2019). International Consensus Guidelines for the Optimal Use of the Polymyxins: Endorsed by the American College of Clinical Pharmacy (ACCP), European Society of Clinical Microbiology and Infectious Diseases (ESCMID), Infectious Diseases Society of America (IDSA), International Society for Anti-infective Pharmacology (ISAP), Society of Critical Care Medicine (SCCM), and Society of Infectious Diseases Pharmacists (SIDP). Pharmacotherapy.

[27] Vardakas KZ, Falagas ME (2017). Colistin versus polymyxin B for the treatment of patients with multidrug-resistant Gram-negative infections: a systematic review and meta-analysis. Int J Antimicrob Agents.

[28] Colistin for lung infection: an update | Journal of Intensive Care | Full Text [Internet]. [cited 2023 Aug 5]. Available from: https://jintensivecare.biomedcentral.com/articles/10.1186/s40560-015-0072-9

